# Transcriptomic and enzymatic analysis reveals the roles of glutamate dehydrogenase in *Corynebacterium glutamicum*

**DOI:** 10.1186/s13568-022-01506-7

**Published:** 2022-12-28

**Authors:** Fanglan Ge, Jingkun Sun, Yao Ren, Bing He, Jiao Li, Sen Yang, Wei Li

**Affiliations:** 1 College of Life Sciences, Sichuan Normal University, Chengdu, 610068 People’s Republic of China; 2 Pingdingshan University, Pingdingshan, 467000 People’s Republic of China

**Keywords:** *Corynebacteriumglutamicum*, Glutamate dehydrogenase, RNA-Seq analysis, Enzymatic characteristics, Nitrogen metabolism

## Abstract

Glutamate dehydrogenase (Gdh), catalyzing the reversible conversion between 2-oxoglutarate and glutamate, plays an important role in the connection of nitrogen and carbon metabolism. Yet little is known about these enzymes in the amino acid-manufacturing *Corynebacterium glutamicum*. In the present study, we firstly identified the enzymatic characteristics of two Gdhs (GdhA and GdhB). The results showed that both GdhA and GdhB prefers NADPH as a coenzyme and have higher affinity for 2-OG than glutamate. The growth characteristics of *gdhA*Δ mutant and *gdhB*Δ mutant, *gdhAB*Δ mutant showed GdhA serves as the main conduit for ammonium assimilation, and GdhB is the main glutamate- metabolizing enzyme in *C. glutamicum*. The full-genome transcriptomic analysis was used to investigate physiological response of *C. glutamicum* to the glutamate as nitrogen source, and *gdh* deletion. The results showed that the nitrogen starvation response was elicited when glutamine served as the sole nitrogen source. *gdhAΔBΔ* double deletion trigger a partially deregulated nitrogen starvation response, in which genes involved in nitrogen assimilation showed obviously upregulated in a certain extent. On the other hand, the genes of phosphotransferase system (PTS) and glycolysis pathway, most genes in pentose phosphate pathway were significantly upregulated, indicating that *gdh* deficiency initiated the enhancement of the absorption and metabolism of carbon sources. We believed that our results in this study will give new insights on the molecular mechanism of Gdh activity cross-talks with carbon and nitrogen metabolism, also setting a new background for further flux redistribution applied research of biotechnological interest.

**Supplementary Information:**

The online version contains supplementary material available at 10.1186/s13568-022-01506-7.

## Introduction

*Corynebacterium glutamicum*, as a non-pathogenic, soil-derived gram-positive actinobacterium, is used as a safe industrial producer of various amino acids (especially L-glutamate, L-lysine and L-arginine), nucleotides, and organic acids (Tsuge et al. 2021; Sheng et al. 2021; Ge et al. 2021). Furthermore, the availability of genetic engineering methods and easy cultivation has helped to make *C. glutamicum* as a model organism and a cell factory of choice in industrial biotechnology (Wang et al. 2021; Mei et al. 2016).

**Fig. 1:**
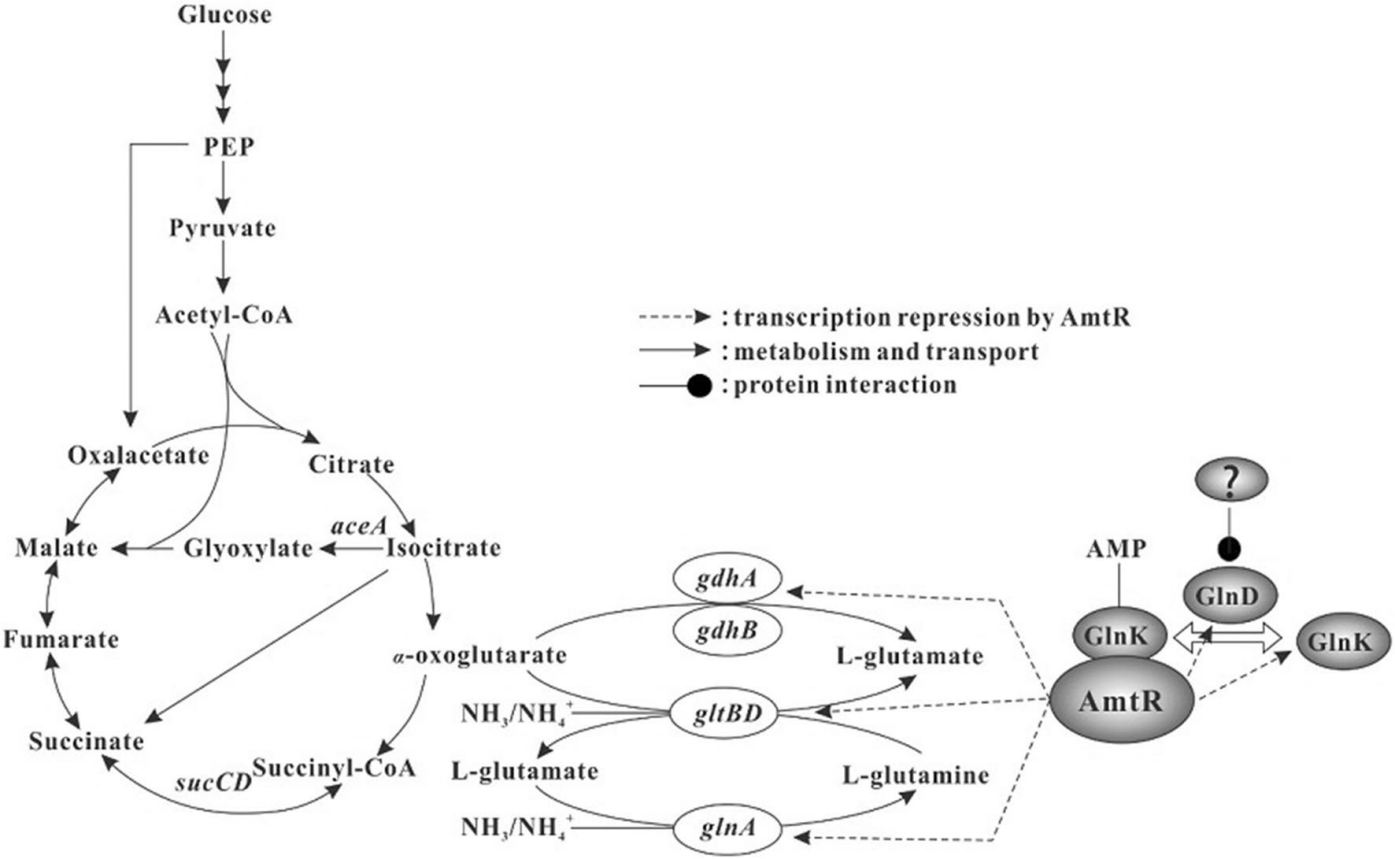
Ammonium assimilation and AmtR regulation in *C. glutamicum* Description of all genes has been given in Additional file 1: Table S1

Carbon and nitrogen are essential components for microbial growth. Glycolysis, the pentose phosphate pathway and tricarboxylic acid cycle (TCA), as the main pathways of carbon metabolism, are not only crucial for the generation of energy, biomass but also for the production of high added-value metabolites (Kobayashi et al. 2020) (see Fig. 1). Maintaining proper intracellular carbon and nitrogen levels is important in cell physiology to maximize nutrient utilization, which implies that nitrogen assimilation must constantly keep pace with carbon utilization, but the underlying mechanism remains poorly understood (Forchhammer et al. 2020).

Many attempts have been made to understand mechanism for nitrogen metabolism and its regulation in *C. glutamicum* (Nolden et al. 2001b; Burkovski et al. 2007; Xu et al. 2020). Ammonium, a preferred nitrogen source of most bacteria, can be assimilated by *C. glutamicum* via two metabolism pathways (see Fig. 1). The longer ammonium-assimilating pathway involves two key reactions: the conversion of L-glutamine and 2-oxoglutarate to two molecules of L-glutamate by glutamate synthase (GOGAT), and the ATP-dependent conversion of L-glutamate and ammonia to L-glutamine by glutamine synthetase (GS). In the shorter pathway, 2-oxoglutarate and ammonium are directly converted to glutamate by the NADPH-dependent glutamate dehydrogenase (Gdh). The other nitrogen metabolism-related genes encoding transporters and enzymes for ammonium assimilation (*amtA, amtB, glnA, gltBD, gdh, glnA*), and urea (*urtABCDE, ureABCEFGD*) metabolism, as well as signal transduction proteins (*glnD, glnK*) are tightly regulated by a TetR-family protein AmtR, which blocks transcription of those genes during growth in nitrogen-rich medium (Grau et al. 2021; Buchinger et al. 2009; Rehm et al. 2010). Under conditions of nitrogen limitation, GlnK is adenylylated by adenylyltransferase GlnD, and interacts with AmtR, engendering the dissociation of AmtR from its target promoters (Grau et al. 2021; Silberbach et al. 2005).

Schmid et al. demonstrated that the nitrogen starvation improves the transcription of genes encoding glycolysis enzymes in *C. glutamicum* (Rehm et al. 2010). In Cyanobacteria, nitrogen depletion upregulated the expression of genes encoding glutamate synthases (*gltD* and *gltB*) as well as accumulation of metabolites in glycolysis (fructose-6-phosphate, fructose-1,6-bisphosphate, and glyceraldehyde-3-phosphate) and TCA cycle (Schmi et al. 2000; Joseph et al. 2014).

Glutamate dehydrogenase (Gdh), catalyzing the reversible conversion between 2-oxoglutarate/ammonium and glutamate, is a key enzyme connecting TCA and amino acid metabolism, and plays important role in the balance of nitrogen and carbon homeostasis in cells (Jayaraman et al. 2022; Hänssler et al. 2009; Sieg et al. 2014). In some *C. glutamicum* strains, there is only one NADP specific glutamate dehydrogenase gene in their genomes, while others contain two putative glutamate dehydrogenase genes: a NADPH specific glutamate dehydrogenase gene, and a NAD^+^ /NADP^+^ dual-specific glutamate dehydrogenase gene. Interestingly, industrial amino acid producing *C. glutamicum* strains tend to have two functional *gdh* genes (Rehm et al. 2010; Yang et al. 2017).

However, the information on of Gdh in nitrogen and carbon remains largely unavailable. It have been reported that glutamate dehydrogenase gene exhibits constitutive expressing in different media, and *gdh* mutation elicits a partially deregulated nitrogen starvation response in *C. glutamicum* using RNA hybridization, showing that Gdh activity is involved in nitrogen metabolism (Schmi et al. 2000; Hänssler et al. 2009) DNA microarray technology was used to investigate the transcriptomic response of *C. glutamicum* grown in different nitrogen sources (Rehm et al. 2010), the results showed that growth with L-glutamine as the sole nitrogen source elicited the nitrogen starvation response.

NADP^+^ preference has been identified in *C. glutamicum* ATCC 13,869 and 13,032. The starting strain *C. glutamicum* F343 was derived from *C. glutamicum* S9114 (Yang et al. 2017; Zhang et al. 2018), which is commonly used for the industrial production of glutamate, and there are two putative *gdh* genes (*gdhA*, *gdhB*) in the genome. In our previous study, deletion of the *gdhA* reduced glutamate yield and draw the flux of the TCA cycle to heme pathways.

In this work, to understand how Gdh cross-talks with carbon and nitrogen metabolism of *C. glutamicum*, the *gdh* single and double knockout mutants were constructed firstly. Then the biochemical and physiological characterization of the two Gdh were investigated. In addition, for the first time, full-transcriptomic changes of *C. glutamicum* was compared when it grows on L-glutamate or ammonium as a sole nitrogen source, and also the effect of *gdh* gene deletion on the expression of genes involve in TCA cycle, glycolysis pathway, ammonium assimilation, glucose phosphotransferase system (PTSGlc) were analysed, attempting to give new insights on the molecular mechanism of Gdh activity cross-talks with carbon and nitrogen metabolism, also offering new understanding for further flux redistribution applied research of biotechnological interest.

## Materials and methods

### Bacterial strains and culture conditions

Details on the bacterial strains and plasmids which were used in this study are listed in the Table 1, while oligonucleotide sequences are presented in Additional file 1: Table S1. *Escherichia coli* strains DH5α and BL21 (DE3) were stored in the laboratory, and used for gene cloning and expressing purposes, respectively. *Corynebacterium glutamicum* strain F343 (Zheng et al. 2012), gifted by Professor Pu Zheng of Jiangnan University, was used as the parent strain. The plasmid pK18mobsacB (Schäfer et al. 1994) was used for gene disruption in strain F343. Plasmid pXMJ19 was used to over-express genes in *C.glutamicum*. LBHIS medium, containing 2.5 g/L yeast extract, 5 g/L tryptone, 5 g/L NaCl, 18.5 g/L brain heart infusion, and 91 g/L sorbitol was used for the electrophoretic transformation of *C. glutamicum*. The seed medium contained 20 g/L glucose, 3 g/L corn steep liquor, 5 g/L yeast extract, 10 g/L tryptone and 10 g/L NaCl.

**Table 1:** Strains and plasmids used in this study

Strains and plasmids	Characteristic(s)	Sources
Strain F1	Wild type *C.glutamicum* F343	Zheng et al. 2012
Strain F2	*C.glutamicum* F343 ∆*gdhA*	Ge et al. 2021
Strain F3	*C.glutamicum* F343 ∆*gdhB*	This study
Strain F4	*C.glutamicum* F343 ∆*gltB*	This study
Strain F5	*C.glutamicum* F343 ∆*gdhA*∆*gdhB*	This study
Strain F6	*C.glutamicum* F343 ∆*gdhA*∆*gdhB*∆*gltB*	This study
pK18mobsacB	Mobilizable *E.coli* cloning vector, Km^r^	Kvitko et al. 2011
pK18mobsacB-∆*gdhB*	Integrative transformation vector for deletion of the *gdhB* gene	This study
pK18mobsacB-∆*gltB*	Integrative transformation vector for deletion of the *gltB* gene	This study
*E.coli* DH5α	Wild-type strain; subcloning host	Laboratory stock
*E.coli* TSBL21 (DE3) *pLysS*	λDE3 lysogenic bacteria, with T7 RNA polymerase and genes expressing T7 lysogenic bacteria	Laboratory stock
*E.coli* BL21 (DE3)	Lysogenic λDE3 bacteria with T7 RNA polymerase	Laboratory stock
*E.coli* TS setta (DE3)	With six rare codon AUA, AGG, AGA, CUA, CCC, and GGA corresponding TRNA	Laboratory stock
pET28a( +)	*E.coli* expressing vector, *Km*^*r*^	Laboratory stock
pET28a( +)-*gdhA*	pET28a( +) harboring the *gdhA* gene	This study
pET28a( +)-*gdhB*	pET28a( +) harboring the *gdhB* gene	This study

Colonies from a fresh LB agar plate (Sambrook et al. 2001) were inoculated into 50 ml seed medium in a 500 ml baffled shake flask and incubated overnight at 30 ℃ and 200 r.p.m, the cells were harvested by centrifugation for 5 min at 6000 r.p.m and 4 ℃, washed with 0.9% normal saline without any carbon or nitrogen source, then the cells were inoculated into 50 mL of a modified CGXII medium (Keilhauer et al. 1993) which contained 70 mM glucose as a carbon source and either 70 mM glutamate or 150 mM ammonium sulfate as a sole nitrogen source in 500 mL conical flasks, with an initial OD_600_ nm of 0.1, and were incubated for the indicated times at 30 ℃ and 220 r.p.m. For the transcriptome comparison, the cultures were grown for 24 h, and cells were harvested and used for RNA preparation.

### Preparation of recombinant GdhA and GdhB

GdhA and GdhB were prepared as described below. *gdhA*(C629_ RS10180), *gdhB* (C629_RS14595) were amplified by polymerase chain reactions (PCR) using the genome DNA of *C. glutamicum* F343 as a template with the primers gdhAF, gdhAR, gdhBF, gdhBR, respectively. After the DNA sequence was confirmed, the fragments were ligated with the expression vector pET28a to yield pET28a-*gdhA*, pET28a-*gdhB* respectively. *E. coli* cells harboring pET28a-*gdhA*, pET28a-*gdhB* were grown in 50 mL of LB medium supplemented with 20 µg/ mL Kanamycin, cultured at 37 ℃, 220 rpm until the OD_600_ was 0.6–0.8, and IPTG was added to the final concentration of 0.2 mmol/L; Expression was induced at 18 ℃ for 12 h. The cells harvested by centrifugation at 10,000 g at 4 °C for 10 min were washed, suspended in 20 mM Tris–HCl (pH 8.0), and disrupted with an ultrasonic disruptor (Sonics& Materials, Inc., Newtown, CT, USA) under ice bath conditions. Cell debris was removed by centrifugation at 40,000 g at 4 °C for 20 min. The supernatant was applied to a Ni-NAT affinity chromatography column (QIAGEN, Hilden, Germany), pre-equilibrated with 20 mM Tris–HCl (containing 150 mM NaCl, and 20 mM imidazole, pH 8.0) at 4 °C. After washing the column with the same buffer, the proteins adsorbed to the resin were eluted with 20 mM TrisHCl (pH 8.0), containing 150 mM NaCl, and 500 mM imidazole, The Gdhs were concentrated using VIVASPIN-20 centrifugal filtration with a molecular 10 cut-off, and detected by 10% SDS-PAGE gel electrophoresis.

### Enzyme assay

To determine the reductive amination activity of Gdh, the reaction mixture contained 50 mM Tris–HCl (pH 7.0), 5 mM mercaptoethanol, 40 mM ∝- ketoglutarate, 90 mM NH4Cl and 0.3 mM NADPH/NADH (Tomita et al. 2017). To determine the oxidative deamination activity of Gdh, the reaction mixture contained 50 mM Tris–HCl, 5 mM mercaptoethanol, 120 mm sodium L-glutamate and 0.5 mM NADP/NAD. The reaction was started by the addition of an appropriate amount of recombinant Gdh solution. The reduction of NAD(P)^+^ to NAD(P)H or oxidation of NAD(P)H to NAD(P)^+^ was monitored at 340 nm in a Shimadzu UV2000 spectrophotometer (Kyoto, Japan). The activity unit of an enzyme is defined as the formation/ consumption of 1 µm NADPH/NADH per minute at 25 ℃. The kinetic parameters (K_m_, V_max_ and k_cat_) of the purified enzymes were determined under the optimum conditions.

### Construction of deletion mutants

The *gdhA* knockout plasmid pK18mobsacB-∆*gdhA* was constructed in our previous study[3]. For constructing *gdhB* knockout plasmid, the primer pair gdhB-U F/R was used to amplify the upstream homologous arm of *gdhB* from *C. glutamicum* F343. The PCR product was digested with *Bam*HI *and Sal*I, and ligated into the suicide vector pK18mobsacB digested with the same enzymes, to generate the plasmid pK18mobsacB-*gdhB*U. Similarly, after using the primer pair gdhB-D F/R to amplify the downstream homologous arm o*f gdhB* from *C. glutamicum* F343, the PCR product was digested with *Sal*I and *Hin*dIII, and ligated into pK18mobsacB -*gdhB*U that was digested with the same enzymes, to yield the plasmid pK18mobsacB-∆*gdhB*.

Transformation of *C. glutamicum* F343 through electroporation was performed as described by the method of Tauch et al. (Tauch et al. 2002). Chromosomal disruption of *gdh*, obtained via the selection of the first and second recombination events, was carried out as described by Schäfer et al. (Schäfer et al. 1994), The kanamycin sensitive colonies resulting from a double crossover event were selected and confirmed by nucleotide sequencing with the primers CheckgdhB F and CheckgdhB R (see Additional file 1: Table S1). The mutant strain was designated as F343-∆*gdhB*. Similarly, using the *gdhA* knockout strain F343-∆*gdhA* as start strain, *gdhA* and *gdhB* double disruption mutant was obtained and designated as F343-∆*gdhA*-∆*gdhB*.

### Transcriptomic analysis

Total RNA was extracted from *C. glutamicum* F343, *C. glutamicum* strain *gdhA*∆*B*∆ grown in medium which contained either 70 mM glutamate or 150 mM ammonium sulfate as a sole nitrogen source, using an RNeasy mini kit (QIAGEN). RNA samples were then treated with RNase Free DNase Set (QIAGEN) and Ribo-Zero rRNA Removal Kit (Epicentre Biotechnologies) to remove any genomic DNA and rRNA respectively. RNA was fragmented and used as a template for PCR using random primers. Strand-specific cDNA libraries were prepared with the mRNA-seq Sample Prep kit (Illumina) and the libraries were sequenced on an Illumina NovaSeq 6000 platform (Novogene, Beijing). The sequence data was deposited to the NCBI Sequence Read Archive (SRA, https://www.ncbi.nlm.nih.gov/sra) with the accession number PRJNA872888. The raw reads were subsequently filtered to prepare clean reads (the result of RNA-Sequencing read mapping of different samples was showed in Additional file 1: Table S2). SOAPaligner/SOAP2 (Li et al. 2010) was used to align the reads to the reference *C. glutamicum* SCgG2 genome sequence (Accession number: NC_021352.1). Expression levels were calculated by FPKM (fragments per kilobase of transcript per million fragments mapped) (Li et al. 2011). Differentially expressed genes (DEGs) analysis was carried out on the DESeq package described by Anders and Huber (Anders et al. 2010) (Statistics of differentially expressed genes of different samples were showed in Additional file 1: Table S3) and KEGG pathway enrichment analysis of the DEGs was implemented in KOBAS software (v2.0.12) (Mao et al. 2005) to test the statistical enrichment of DEGs in KEGG pathways. Gene Ontology (GO) enrichment analysis of the DEGs was conducted by the GO seq (Young et al. 2010).

## Results

### Enzymatic characteristic analysis of GdhA and GdhB

In the genome of *C. glutamicum* F343, both C629_RS10180 and C629_RS14595 were annotated as NADP-specific glutamate dehydrogenase. The former shows 99% homologous to the identified *gdhA* of *C. glutamicum* ATCC 13869 at amino acid level (Tomita et al. 2017). While the latter, sharing a low homologous (26%) to *gdhA* at the amino acid level, was designated *gdhB*. To evaluate their functions involved in nitrogen metabolism, we firstly investigated the enzymatic properties of them. *gdh A*, *gdhB* have been successfully expressed and purified to above 95% purity (see Additional file 1: Fig. S1).

The kinetic parameters of GdhA and GdhB in reductive amination and oxidative deamination reaction were investigated, respectively (see Tables 2 and 3). When NADPH and NH_4_Cl were used as the substrates, the kinetic parameters (K_m_ and V_max_) of GdhA for 2-oxoglutarate (2-OG) were 2.534 mm/L and 0.809 IU/mg, respectively. While the substrate was replaced by NADP, the K_m_ and V_max_ values of glutamate were 87.428 mm/L and 0.544 IU/mg, indicating that GdhA has higher affinity for 2-OG than glutamate (see Table 2). While k_cat_/K_m_ value of 2-OG (3.193 mmol^−1^ min^−1^) was approximately 61–fold that of glutamate (0.0311 mmol^− 1^ min^− 1^), implying that GdhA can catalyze the reaction of glutamate synthesis more efficiently than that of glutamate degradation. In addition, the K_m_ value (0.166 mmol/L) for NADPH was threefold lower than that (0.505 mmol/L) for NADP (Table 2). k_cat_/K_m_ value of NADPH (92.655 3 mmol^− 1^ min^− 1^) for the reaction using NADPH as a coenzyme was -fold higher than that (26.554 mmol^− 1^ min^− 1^) for the reaction using NADP (Table 2), indicating that GdhA was highly specific to NADPH.

**Table 2:** Kinetic parameters of GdhA for reductive amination and oxidative deamination reaction

Reaction	Substrate	K_m_ $(\text{ mmol/L})$	V_max_ $(\text{ IU/mg})$	K_**cat**_ $({\text{mi}}{\text{n}}^{-1})$	K_cat_/K_m_ (min^− 1^ L mmol^− 1^)
Reductive amination	NADPH	0.166	1.539	15.39	92.655
	∝-KG	2.534	0.809	8.09	3.193
Oxidative deamination	NADP	0.505	1.341	13.41	26.554
	glutamate	174.856	0.544	5.44	0.0311

**Table 3:** Kinetic parameters of GdhB for reductive amination and oxidative deamination reaction

Reaction	Substrate	K_m_ $(\text{ mmol/L})$	V_max_ $(\text{ IU/mg})$	K_cat_ $({\text{mi}}{\text{n}}^{-1})$	K_cat_/K_m_ $({\text{min}}^{-1}\cdot \mathrm{L}\cdot {\text{mmol}}^{-1})$
Reductive amination	NADPH	0.0382	1.539	26.61	696.779
	α-KG	4.109	0.809	9.77	2.378
Oxidative deamination	NADP	0.412	1.341	6.42	15.581
	glutamate	47.378	0.544	2.77	0.0586

With regard to GdhB, the kinetic parameters (K_m_ and V_max_) for 2-oxoglutarate (2-OG) were 4.109 mm/L and 0.977 IU/mg, respectively, while the *Km* and V_max_ values of glutamate were 47.378 mm/L and 0.277 IU/mg, indicating that GdhB also has higher affinity for 2-OG than glutamate (see Table 3). The K_m_ value (0.0382 mmol/L) of GdhB for NADPH was 82–fold lower than that (3.133 mmol/L) for NADH, suggesting that GdhB also was highly specific to NADPH. Furthermore, k_cat_/K_m_ value of 2-OG (2.378 mmol^− 1^ min^− 1^) was approximately 40–fold that of glutamate (0.0586 mmol^− 1^ min^− 1^), implying that GdhB can catalyze the amination reaction of 2-OG to synthetize glutamic acid, instead of deamination of glutamic acid.

### Phenotypes of GdhA, GdhB and GltBD mutant strains

**Fig. 2:**
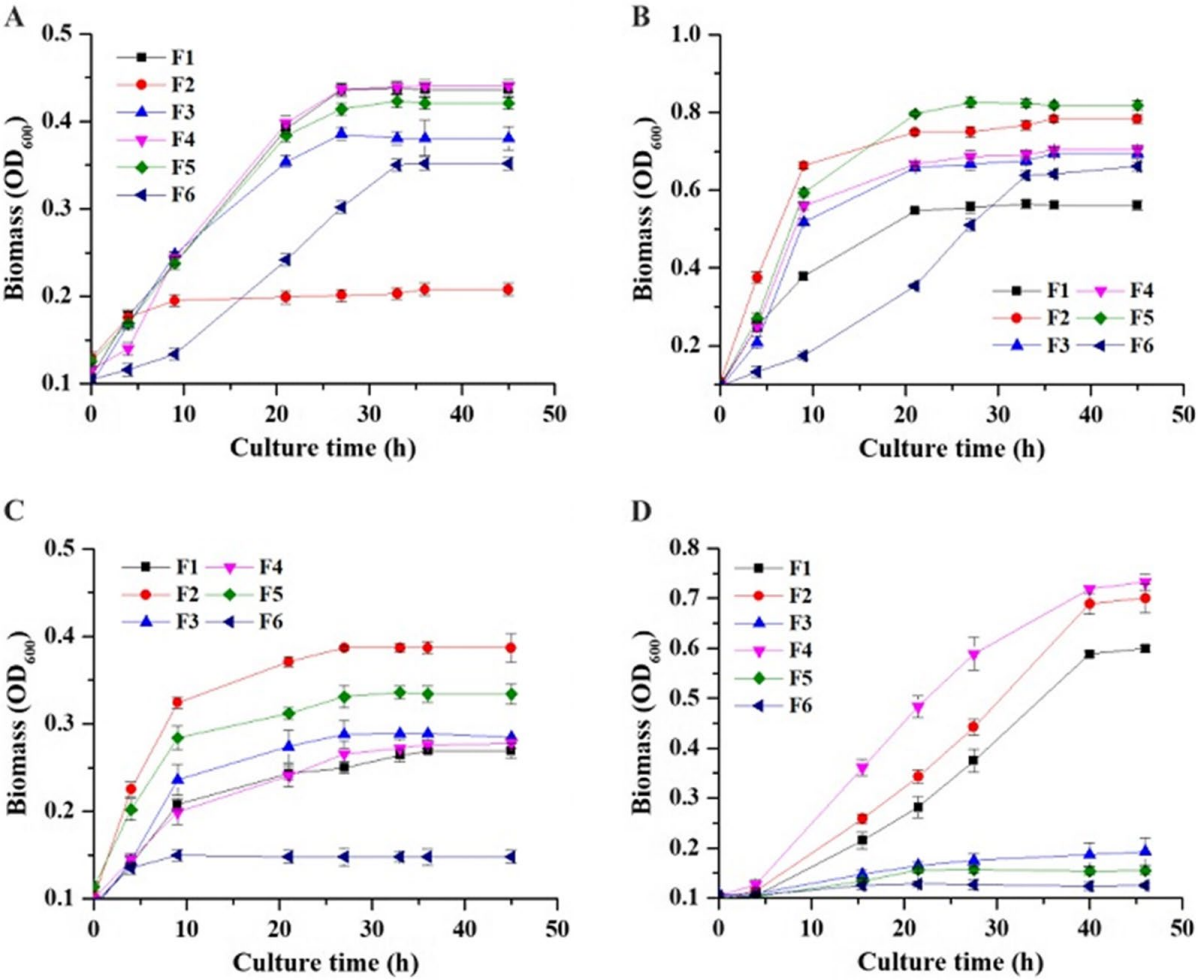
Growth phenotypes of different strains with different nitrogen sources. **A** ammonium, (**B**) glutamine, (**C**) arginine, (**D**) glutamate. Strain denotes: wild type (F1), ∆*gdhA* (F2), ∆*gdhB*(F3), ∆*gltB*(F4), ∆*gdhA*∆ *gdhB*(F5), ∆*gdhA*∆*gdhB*∆*gltB* (F6)

In order to elucidate the cellular roles of these different genes, a series of mutant strains was constructed (details in Table 1) and tested for growth on ammonium or glutamate as the nitrogen source. During growth in CGXII minimal medium with glucose as the carbon source and ammonium sulfate as the nitrogen source, strain F2 lacking GdhA (*ΔgdhA* strain) display impaired growth (see Fig. 2A), however *gdhA* deletions hardly affected the growth with glutamine, arginine and glutamate in comparison with wild type strain (see Fig. 2B–D), implying that GdhA is indispensable for growth with ammonium as sole nitrogen source. Thus, these analyses of growth phenotypes provide clear evidence that GdhA functions in the ammonium assimilation.

When ammonium, glutamine, arginine as nitrogen source respectively, as observed by maximum OD_600_, knockout of GdhB had little effect on growth as compared with wild type strain F1 (see Fig. 2A–C). In addition, using glutamate as nitrogen source, strain F3 showed almost no growth. *gltB* deletion (strain F4) did not affect the growth with several nitrogen sources, different from the previous report which showed that ΔgltB mutant grew normally with ammonium, but exhibited significantly impaired growth on glumine (Rehm et al. 2010).

Unexpectedly, Δ*gdhA*Δ *gdhB* double mutant showed growth in a very different manner. Comparison of the growth of the *gdhAΔ* single mutant with that of the g*dhAΔ gdhBΔ* double mutant (strain F5) on medium with ammonium or glutamine as nitrogen sources reveals that, rather than further impairing growth, the additional loss of GdhB improves growth (see Fig. 2A–C). Meanwhile it showed slower than the wild type strain when using arginine as nitrogen source. However, strain F5 showed almost no growth with glutamate, which was similar to the Δ *gdhBΔ* single mutant strain F3. Compared with *gdhAΔBΔ* double mutant, loss of *gltB* in triple mutant strain F6 significantly affected growth with ammonium or glutamine, and similar to *gdhAΔBΔ* double mutant, strain F5 also showed no growth with glutamate (see Fig. 2D).

### Transcriptome profiling of C. glutamicum F343 grown with ammonium or glutamate as nitrogen

Based on GO classification system, a total of 26 GO terms were enriched with significant (corrected p value < 0.05, log2 ratio ≥ 1), 11 GO terms in biological process, 3 terms in molecular function, and 12 term in cellular component (Additional file 1: Fig. S2). To find the significant metabolic response of strain F343 grown to different nitrogen source, the KEGG database was used to perform enrichment analysis, 615 DEGS were classified into 75 standard KEGG pathways (see Additional file 1: Fig. S3). The maximum number of DEGs (194) was found to be enriched in biosynthesis of secondary metabolites, accounting for 31.54% of the total enriched genes, followed by microbial metabolism in diverse environments. Similarly, biosynthesis of amino acids, carbon metabolism, ribosome, and purine metabolism and ABC transporters in metabolism were highly abundant, with 83, 51, 44, 37 and 35 genes, respectively. Analysis of the pathway data identified 75 pathways related to metabolism, and the highly enriched pathways were two-component system (30), quorum sensing (30), pyruvate metabolism (24), oxidative phosphorylation (17), phosphotransferase system (9) (Fig. 4). In total, 5 pathways were significantly enriched with DEGs (P < 0.05), such as “Ribosome”, “Phenylalanine, tyrosine and tryptophan biosynthesis”, “Biosynthesis of amino acids”, “Biosynthesis of secondary metabolites” (see Additional file 1: Fig. S3).

**Fig. 3:**
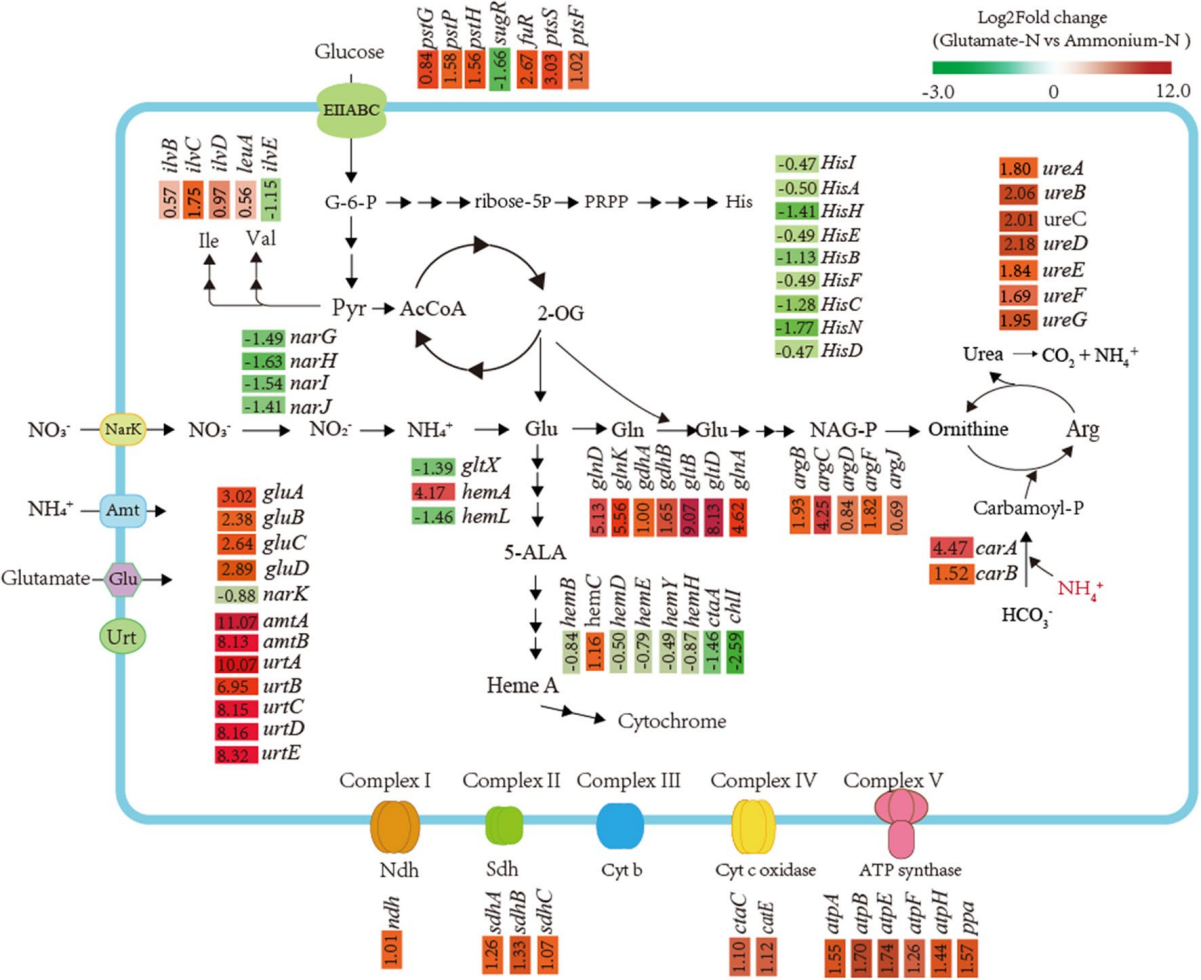
The differentially expressed genes in different metabolic pathways when wild type strain was grown with glutamate instead of ammonium as sole nitrogen source. DEGs in different metabolic pathways are shown using heat maps with gene names. Metabolites and abbreviated according to 13CFLUX2 (www.13Cflux.net) and description of all genes has been given in Additional file 1: Table S4. Red indicates upregulation. Blue indicates downregulation

The most obvious upregulation of genes involving in nitrogen metabolism were observed when *C. glutamicum* F343 grown on glutamate as the sole nitrogen source. Most nitrogen metabolism genes are controlled by negative regulator AmtR. The nitrogen signal transduction protein encoding genes *glnD* and *glnk* were upregulated by 5.13, 5.56 logFC, respectively (see Fig. 3). The genes for ammonium intake, *amtA,amtB*, were 11.07, 8.13-fold upregulated, respectively. While *gdhA* and *gdhB,* encoding glutamate dehydrogenase, were upregulated by1.01, 1.65 fold, the FPKM values from 2818.33, 918.91 raised to 5644.14, 2891.15, respectively. The *glnA* gene, coding glutamine synthetase (GS), was 4.62 -fold upregulated, and the genes of the gltBD operon, encoding the large and the small subunit of glutamate synthase (GOGAT), showed 9.71- and 8.04-fold upregulation, respectively. The urea uptake system *urtABCDE* (Beckers et al. 2001), whose expression was at least sevenfold increased, and the urease operon *ureABCEFGD* (Nolden et al. 2000), whose genes were at least 1.7-fold upregulated. The ABC transporter *gluABCD*, involving in translocation of glutamate, were about threefold upregulated (see Fig. 3). Meanwhile, nitrate transporter-encoding gene nark, nitrate reductase -encoding gene *narGHIJ* were downregulated about onefold.

Some genes involved in the arginine biosynthesis were significantly upregulated (see Fig. 3). Most of genes involved in the isoleucine and valine biosynthesis showed downregulation. At least 11 genes in the in the heme biosynthesis pathway exhibited differential expression, and among them were downregulated, while only *hemA* and hem C showed upregulation (see Fig. 3). As shown in Fig. 3, the transcriptional levels of genes involved in oxidative phosphorylation were upregulated The uptake and phosphorylation of glucose in *C. glutamicum* is mainly through phosphotransferase system (PTS) (Ruan et al. 2020; Tanaka et al. 2008). As shown in Fig. 3, grown on glutamate as sole nitrogen, PTS encoding genes such as *pstG, pstP, pstH, pstS, pstF, fruR* were obviously upregulated, while the repressor-encoding gene sugR was upregulated.

As shown in Fig. 3, the transcriptional levels of genes, including such as *hisA*, *hisB*, *hisC*, *hisD*, *hisF*, *hisH*, *hisI*, *hisN*, involved in biosynthesis pathway of histidine were upregulated. In addition, the genes of serine synthesized from glyceric acid -3-P, including *serA*(C629_RS07285), *serC*(C629_RS05100), *serB*(C629_RS12565), and then *glyA*(C629_RS05820) of glycine synthesized from serine, were upregulated.

### Analysis of transcriptomic changes of C. glutamicum F343 elicited by gdhA gdhB double disruption

When the *gdhA* and *gdhB* double mutant F5 grown under ammonium, compared with the start strain F1, a total of 44 GO terms were enriched with significant (corrected p value < 0.05, log2 ratio ≥ 1), 26 GO terms in biological process, 12 terms in molecular function, and 7 term in cellular component (Additional file 1: Fig. S4). The KEGG database was used to analyses the effect of *gdh* gene disruption on the transcriptome changes. 355 DEGS were classified into 75 standard KEGG pathways (see Additional file 1: Fig. S5). The maximum number of DEGs (122) was found to be enriched in biosynthesis of secondary metabolites, accounting for 34.37% of the total enriched genes, followed by microbial metabolism in diverse environments. Biosynthesis of amino acids, carbon metabolism, ABC transporters, ribosome, glycolysis/ gluconeogenesis, alanine, aspartate and glutamate metabolism were high abundant, with 54, 41, 30, 29, 22, 21 genes, respectively. In total, 16 pathways were significantly enriched with DEGs (P < 0.05), such as “Phosphotransferase system (PTS)”, “Alanine, aspartate and glutamate metabolism”, “Nitrogen metabolisms”, “Carbon metabolism”, “Glycolysis / Gluconeogenesis”, and so on (see Additional file 1: Fig. S5). Therefore, it can be considered to promote the accumulation of amino acid such as alanine, aspartate by knocking out *gdh* gene in metabolic engineering.

**Fig. 4:**
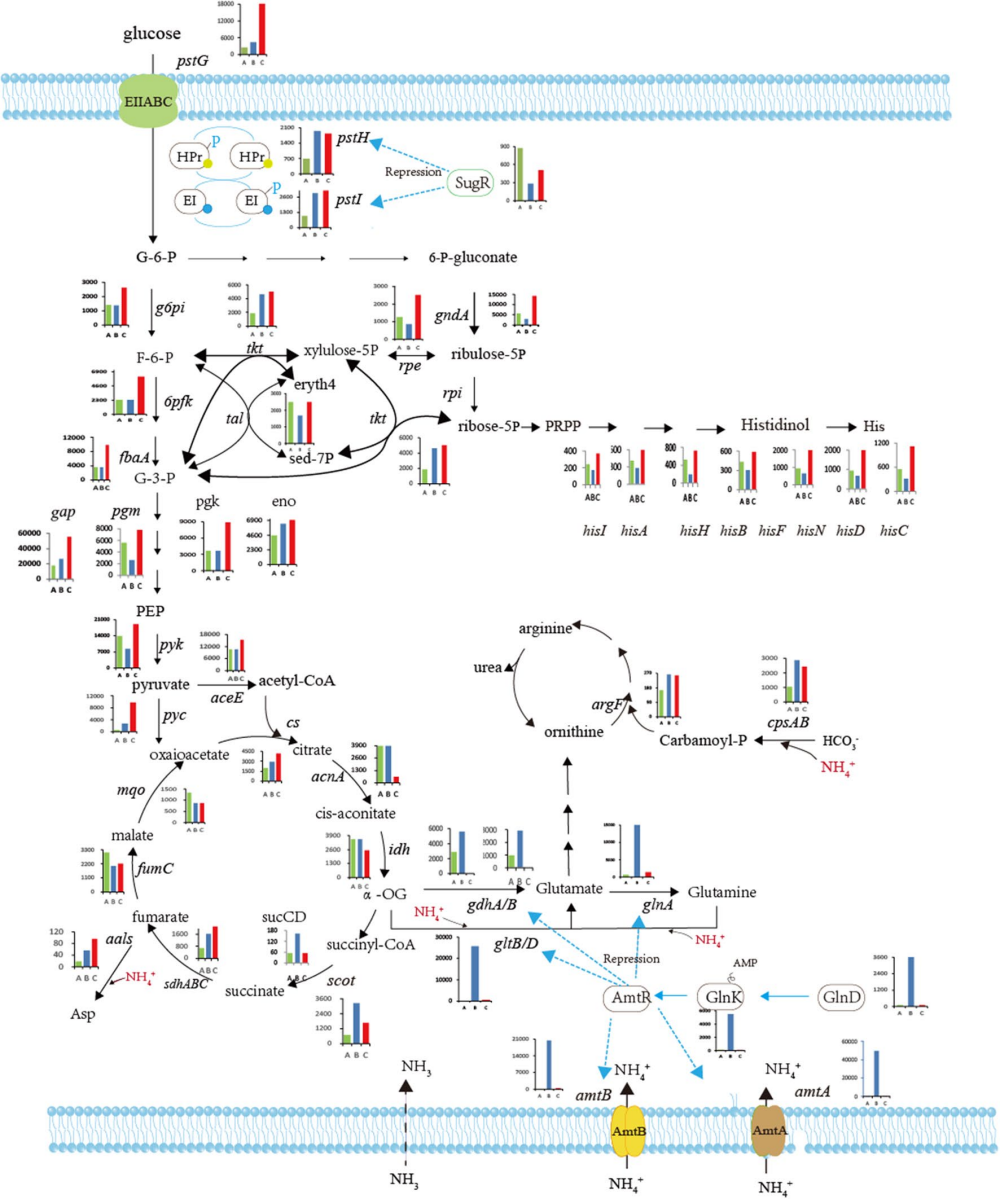
Changes of expression of genes in different metabolic pathways of strains F1 and F5 grown with ammonium. Reactions of the central metabolism together with several biosynthesis pathways are shown here. Metabolites are abbreviated according to 13CFLUX2 (www.13Cflux.net) and description of all genes has been given in Additional file 1: Table S5. The bar graph represents the FPKM value of the gene, and the numbers indicate the values of the expression levels (FPKM) (please see the Martials & Methods). The **A**, **B**, **C** from left to right are: *C.glutamicum* F343 (strain F1) with ammonium, *C.glutamicum* F343 (strain F1) with glutamine, *C.glutamicum* ∆gdhA∆gdhB (strain F5) with ammonium, respectively

DEGs involved in metabolic pathways are presented in Fig. 4. In *C. glutamicum gdh* gene double disruption mutant compared to *C. glutamicum* F1 under ammonium, the genes involved in nitrogen assimilation, including *gltB, gltD, glnA, amtA,amtB* upregulated by 3.85, 2.76, 1.18, 3.29, 2.59 fold, respectively. While no obvious expression changes of *glnD, glnK* were observed (see Fig. 4).

The genes involved in the uptake of sugars (phosphotransferase system) (PTS) were significantly upregulated, similar to that of train F1 grown under glutamate (see Fig. 4). Especially, the FPKM value of *pstG* increased from 4354.08 to 18,166.13. Interesting, all of the genes, including *gap*, *pgk*, *fbaA*, *ldh*, *6pfk*, *eno, pyk*, *g6pi*, *aceE*, *pgm*, involved in the glycolysis, were significantly upregulated. In addition, most of the genes in the pentose phosphate pathway also significantly upregulated*. ΔgdhAΔgdhB* double disruption had relatively complex effect on the expression of the genes in the TCA cycle (see Fig. 4). *acnA, idh, fumC, mqo* were significantly downregulated, and *pyc, sdhA, sdhB, sdhC* were upregulated.

### Analysis of transcriptomic changes of C. glutamicum F343 elicited by gdhB disruption

*gdhB* single disruption had little effect on the transcriptome profiling. When the *gdhB* single mutant grown under ammonium, compared with the start strain F1, a total of 3 GO terms were enriched with significant (corrected p value < 0.05, log2 ratio ≥ 1), the 3 terms were involved in carbohydrate transport, transport and antioxidant activity. The KEGG database was used to perform enrichment analysis, 57 DEGS were classified into 48 standard KEGG pathways (Additional file 1: Fig. S6). In total, 9 pathways were significantly enriched with DEGs (P < 0.05) (Additional file 1: Fig. S6).

In *C. glutamicum gdhB* gene disruption mutant, compared to *C. glutamicum* F1 under ammonium, a significant differential expression of several genes involved in the transport of different metabolites was observed in our experiment (Additional file 1: Table S6). The ABC transporter *gluABCD*, involving in translocation of glutamate, were 1.47, 1.56, 1.73, 1.81-fold upregulated, respectively (Additional file 1: Table S6). And the ribose transporters *rbsA, rbsA, rbsB, rbsC, rbsd* were 1.71, 1.62, 1.51, 1.71-fold upregulated, respectively. In addition, the transcriptional levels of some genes involved in pentose phosphate pathway, were upregulated. Analysis of the transcriptome involved in Oxidative phosphorylation, the transcriptional levels of genes such as *cydA, cydB, ctaA, ctaB, sdhA, sdhB* were upregulated (Additional file 1: Table S6).

## Discussion

When present in high concentration of ammonium, Gdh assimilates primarily ammonium via catalyzing the reductive amination of 2-OG (see Fig. 1), playing a pivotal role in link between carbon and nitrogen metabolism. This is the first in-depth investigation of the role of glutamate dehydrogenase in *C. glutamicum* based on biochemical characterization and genome-wide transcriptomic analysis. Similar to most *C. glutamicum* strains widely used in fermentation industry, *C. glutamicum* F343 genome contain two *gdh* genes: *gdhA* and *gdhB*, they share a low homologous (26%) to each other. The enzymatic characteristics analysis indicated that GdhA prefers NADPH to NADH as a coenzyme and can catalyze the reaction of glutamate synthesis more efficiently than that of glutamate degradation. The K_m_ value for NADPH was 1/7 of that for NADP^+^, indicating that the higher specificity for NADPH is one of the key factors for the preference of GdhA for glutamate production. In addition, GdhA showed low and high K_m_ values for 2-OG (2.5 mM) and glutamate (174.856 mM) in the reactions, respectively. These results coincided well with previous reports for in vitro enzymatic analysis of CgGdh from *C. glutamicum* ATCC 13869 (Tomita et al. 2017), and also were consistent with previous reports for in vivo functional analysis of CgGdh, which revealed that CgGdh contributed to the overproduction of glutamate (Jo et al. 2012; Kholy et al. 1993). The K_m_ value (0.0382 mM) of CgGdhB for NADPH was similar to that of other Gdhs (Tomita et al. 2017), but different from that of *C. glutamicum* S9114, GdhB of which showed higher affinity for NADH (Wang et al. 2003). In addition, the K_m_ value (4.109 mM) for 2-OG was also close to that (9.6 mM) of *E.coli* (Bennett et al. 2009).

When ammonia was supplied as the sole nitrogen source, *gdhA*-deficient strain exhibited significantly impaired growth compared with wild type, paralleling those reported previously (Schäfer et al. 1994). And glutamate, glutamine or arginine used as the sole nitrogen source, respectively, *gdhA*-deficient strain grown as fast as the wild type, similar to a previous report which showed Δ*gdhA* mutant reached wild-type growth rates with glutamine (Schäfer et al. 1994). These results indicate that GdhA serves as the main conduit for ammonium assimilation in *C. glutamicum*. Deficient of GdhB (strain F3) had slight effect on growth with either ammonium or glutamine or arginine as nitrogen source, while strain F3(*gdh B*Δ mutant) showed completely inhibited growth with glutamate as nitrogen source, which completely consistent with its rather high value of k_m_ for glutamate, implying that GdhB is the main glutamate- metabolizing enzyme. The elevated growth rate of the *gdhA*Δ*B*Δ mutant grown with either ammonium or glutamate or glutamine or arginine, however, was quite unexpected. One interpretation of this result is that alternate pathways for nitrogen assimilation and glutamate- metabolism are activated when *gdhA* and *gdhB* were simultaneity deleted (Sieg et al. 2014).

To dissect the role of differential gene expression (DEGs) profiles, a global transcriptomic profiling method was employed for the strain F343 genome that grown under ammonium or glutamate as nitrogen sources. The genes involving in nitrogen metabolism, including *glnD*, *glnk, amtA,amtB*, *glnA*, *gltBD*, *urtABCDE*, *gluABCD* were observed strongly upregulated when *C. glutamicum* F343 grown on glutamate as the sole nitrogen source, in line with the previous reports (Sieg et al. 2014; Mu¨ller et al. 2006) which showed that the nitrogen starvation response was elicited when glutamine served as the sole nitrogen source. On the other hand, though *gdhA* and *gdhB* showed slightly upregulated, they expressed at a relatively high level grown with ammonium, which is compatible with the *gdhA* function of assimilating ammonium. This result corresponds well with the previous results (Hänssler et al. 2009; Schäfer et al. 1994).

To understand the role of *gdhA* and *gdhB* in the carbon and nirtrogen metabolism, the effect of *gdhA gdhB* double deficiency on the global transcriptomic profiling was investigated. The results showed *gdhA*Δ*B*Δ double mutant also elicit significantly transcriptomic changes. The genes including *gltB, gltD, glnA, amtA,amtB,* involved in nitrogen assimilation, showed obviously upregulated, but compared to expression level of strain F343 grown with glutamate, the levels of expression are absolutely very low (see Fig. 4), this result implied that *gdhAgdh*B double deletion trigger a partially deregulated nitrogen starvation response, and very consistent with previous reports which showed *glnA* and *gltBD* were deregulated in *gdh* mutant (Mu¨ller et al. 2006).

GS (*glnA*), catalysing conversion of L-glutamate and ammonia to L-glutamine, was responsible for ammonium assimilation under ammonium limitation or nitrogen starvation (Nolden et al. 2001a). In the absence of Gdh, *glnA* upregulated by 1.2 fold, the FPKM value was upregulated from 628.38 to 1419.80, and this enzyme possesses much higher affinity for ammonium than that of Gdh, it must be fully competent to assimilate ammonium. This provided an explanation for the elevated growth rate of the *gdhA*Δ*B*Δ mutant grown with ammonium.

The reversible conversion reaction of fumarate and ammonia to aspartate, catalysed by spartate ammonia lyase (aspartase), was a possible pathway of ammonium assimilation (Schäfer et al. 1994). In the absence of Gdh, aspartase encoding gene *aals*, was upregulated (see Fig. 4), might compensate partially the function of ammonia assimilation. Another reaction involved in ammonia assimilation is the one catalysed by carbamoyl phosphate synthetase (CPS). Studies have shown that carbamoyl-phosphate synthase can use ammonium as substrate, catalyze ammonium and CO_2_ to consume 2 molecules of ATP, and synthesize carbamoyl-phosphate (Sahay et al. 1998). Although it has a higher affinity for glutamic acid (Sahay et al. 1998). The major subunit of *cpsA* (*C629_RS08890*) of *gdhAB* double knockout strain was upregulated by 1.2 fold, the FPKM value was upregulated from 1029.87 to 2440.119, and the minor subunit of *cpsB* (C629_RS08895) was upregulated by 1.0 fold. It is speculated that after *gdhAB* double knock-out, CPS assimilated ammonium and further synthesized glutamic acid.

Both the genes of phosphotransferase system (PTS) and glycolysis pathway were strongly upregulated. These results confirmed that *gdh* double deficiency initiated the enhancement of the absorption and utilization of carbon sources. There is a possibility that *gdh* double deficiency would be beneficial to the accumulation of pyruvic acid and its various fermentation products such as butanol, malic acid, lactic acid, propionic acid. In the TCA, the genes including *acnA*, *idh* upstream of 2-OG were strongly downregulated, suggesting *gdh* deletion blocked the synthesis of glutamic acid and caused reduction of the carbon flux to 2-OG by re-allocating the TCA carbon flux (Ge et al. 2021).

In conclusion, the present study demonstrated that GdhA is a NADPH-dependent enzyme which serves as the main conduit for ammonium assimilation, while GdhB is also a NADPH- preferred enzyme which participate in glutamate metabolism in *C. glutamicum*. As a whole, *gdhAΔBΔ* double mutant elicit significantly transcriptomic changes. Firstly, a partially deregulated nitrogen starvation response was observed. Secondly, expression of *glnA,* aals and *CpsAB* exhibited upregulated, might undertake assimilate ammonium and compensate the lack of Gdh in *gdh*-null mutant with ammonium as nitrogen source. Thirdly, the genes of PTS and glycolysis pathway, most genes in pentose phosphate pathway were significantly upregulated, indicating that *gdh* double deficiency initiated the enhancement of the absorption and metabolism of carbon sources. These results proved clearly that glutamate dehydrogenase have wide cross-talks with carbon and nitrogen metabolism, and play important roles in improving accumulation of metabolites in industrial strains.

## Supplementary Information

**Additional file 1: Table S1.** The primers are designed in this paper. **Table S2.** RNA-Sequencing read mapping. L-glutamate (LG) and ammonium sulfate (S) correspond to two different conditions: 50 mL minimal medium plus with 150 mM ammonium sulfate, 70mMl L-glutamate, respectively. **Table S3.** Statistics of differentially expressed genes with different screening thresholds. **Table S4.** Description of genes in which response to nitrogen form. **Table S5.** Description of genes in which response to *gdh* gene deletion. **Table S6.** The differentially expressed genes involved in metabolism by comparative transcriptomic analysis of strains F3 vs F1. **Figure S1.** Expression and purification of GdhA and GdhB (A) M, protein marker; Lane 1, cell lysate of IPTG induced *E.coli* Tssetta (DE3) carrying pET28a gdhA; Lane 2, purified GdhA. (B) M, protein marker; Lane 1, cell lysate of IPTG induced *E.coli* Tssetta (DE3) carrying pET28a gdhB; Lane 2, purified GdhB. **Figure.S2.** The Gene-Ontology terms and pathway enrichment analysis of differentially expressed genes of F1 strain gown with glutamate instead of ammonium. **Figure S3.** The KEGG pathway analysis of differentially expressed genes of F1 strain gown with glutamate instead of ammonium. **Figure S4**. The Gene-Ontology terms and pathway enrichment analysis of differentially expressed genes of strains F1 vs F5 grown with ammonium. **Figure S5.** The KEGG pathway analysis of differentially expressed genes of strains F5 vs F1 grown with ammonium. **Figure S6**. The KEGG pathway analysis of differentially expressed genes of strains F3 vs F1 grown with ammoniumig.S6 The KEGG pathway analysis of differentially expressed genes of strains F3 vs F1 grown with ammonium.

## Data Availability

The data generated or analyzed during this study are included in this published article and its Additional file.

## Abbreviations

Gdh: Glutamate dehydrogenase
PTS: Phosphotransferase system
TCA: Tricarboxylic acid cycle
GOGAT: Glutamate synthase
GS: Glutamine synthetase
GO: Gene ontology function database
KEGG: Kyoto encyclopedia of genes and genomes pathway database
DEG: Diferentially expressed gene
2-OG: 2-Oxoglutarate
TCA: Tricarboxylic acid cycle
HPr: Histidine protein
PTSGlc: Glucose phosphotransferase system
PCR: Polymerase chain reactions
F_S: *C.glutamicum* F343 grew on ammonium
F_LG: *C.glutamicum* F343 grew on glutamine
FQB_S: *C.glutamicum* ∆GdhA∆gdhB grew on ammonium
FQAB_S: *C.glutamicum* ∆GdhA∆gdhB grow on ammonium
FPKM: Fragments per kilobase of exonmodelper million mapped fragments
