# Supplementary material for: Transcriptomic and enzymatic analysis reveals the roles of glutamate dehydrogenase in Corynebacterium glutamicum
Source: AMB Express. 2022 Dec 28;12:161. doi: 10.1186/s13568-022-01506-7 (PMC9797636; doi:10.1186/s13568-022-01506-7)
Supplement: Supplementary file 1 — Additional file 1: Table S1. The primers are designed in this paper. Table S2. RNA-Sequencing read mapping. L-glutamate (LG) and ammonium sulfate (S) correspond to two different conditions: 50 mL minimal medium plus with 150 mM ammonium sulfate, 70mMl L-glutamate, respectively. Table S3. Statistics of differentially expressed genes with different screening thresholds. Table S4. Description of genes in which response to nitrogen form. Table S5. Description of genes in which response to gdh gene deletion. Table S6. The differentially expressed genes involved in metabolism by comparative transcriptomic analysis of strains F3 vs F1. Figure S1. Expression and purification of GdhA and GdhB (A) M, protein marker; Lane 1, cell lysate of IPTG induced E.coli Tssetta (DE3) carrying pET28a gdhA; Lane 2, purified GdhA. (B) M, protein marker; Lane 1, cell lysate of IPTG induced E.coli Tssetta (DE3) carrying pET28a gdhB; Lane 2, purified GdhB. Figure.S2. The Gene-Ontology terms and pathway enrichment analysis of differentially expressed genes of F1 strain gown with glutamate instead of ammonium. Figure S3. The KEGG pathway analysis of differentially expressed genes of F1 strain gown with glutamate instead of ammonium. Figure S4. The Gene-Ontology terms and pathway enrichment analysis of differentially expressed genes of strains F1 vs F5 grown with ammonium. Figure S5. The KEGG pathway analysis of differentially expressed genes of strains F5 vs F1 grown with ammonium. Figure S6. The KEGG pathway analysis of differentially expressed genes of strains F3 vs F1 grown with ammoniumig.S6 The KEGG pathway analysis of differentially expressed genes of strains F3 vs F1 grown with ammonium. [file 13568_2022_1506_MOESM1_ESM.docx]

**AMB Express**

**Transcriptomic and enzymatic characteristics analysis reveals the roles of glutamate dehydrogenase in nitrogen metabolism of *Corynebacterium glutamicum***

Fanglan Ge^1^*, Jingkun Sun^1,2^*, Yao Ren^1^, Jiao Li^1^, Sen Yang^1^, Wei Li^1^****

College of life Sciences, Sichuan Normal University, Chengdu, 610068, P. R. China^1^

Pingdingshan university, Pingdingshan, 467000, P. R. China ^2^

**These authors contributed equally to this work.

****Corresponding author, Wei Li, [liwei001@sicnu.edu.cn](mailto:weelee201@aliyun.com)


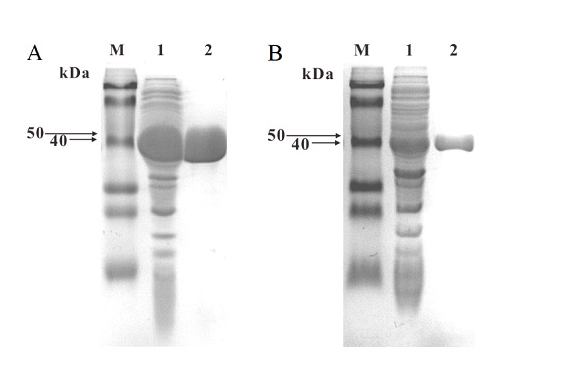


Fig.S1 Expression and purification of GdhA and GdhB (A) M, protein marker; Lane 1, cell lysate of IPTG induced *E.coli* Tssetta (DE3) carrying pET28a gdhA; Lane 2, purified GdhA. (B) M, protein marker; Lane 1, cell lysate of IPTG induced *E.coli* Tssetta (DE3) carrying pET28a gdhB; Lane 2, purified GdhB


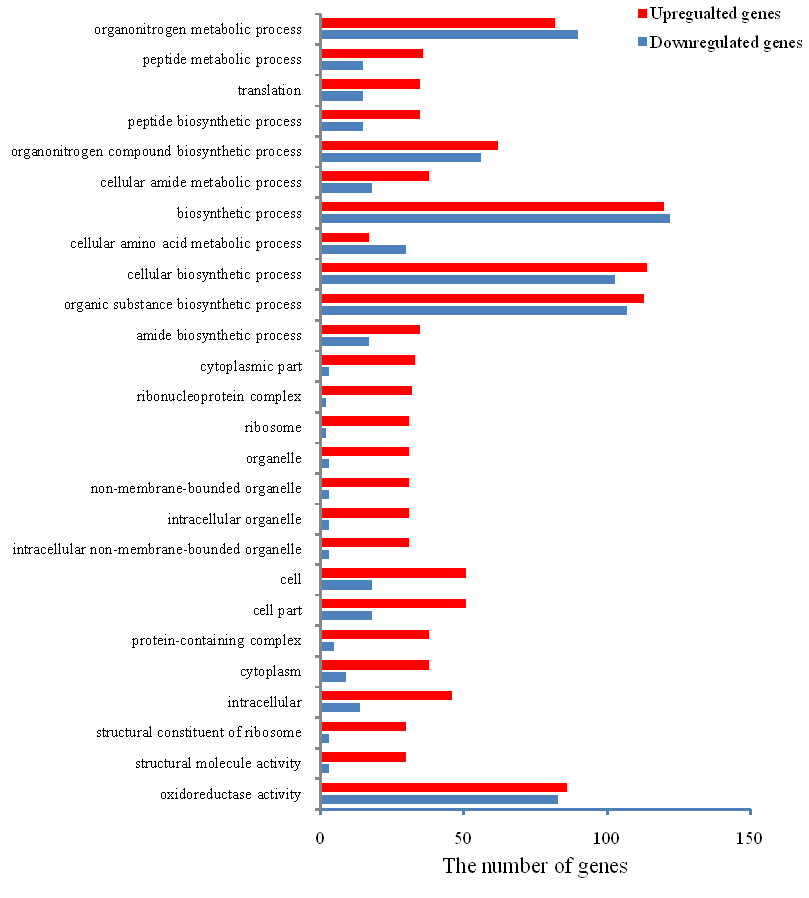


Fig.S2 The Gene-Ontology terms and pathway enrichment analysis of differentially expressed genes of F1 strain gown with glutamate instead of ammonium


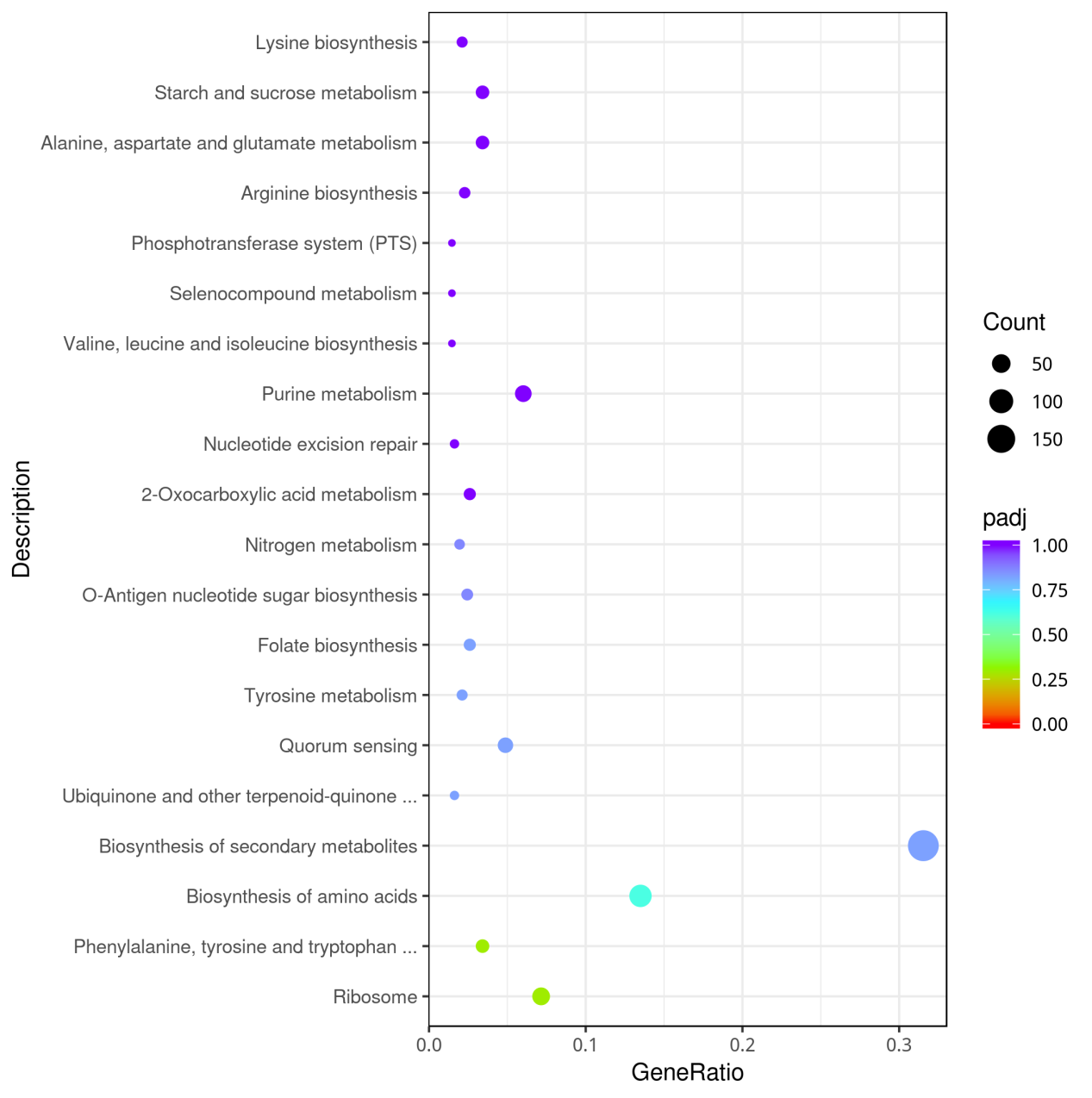


Fig.S3 The KEGG pathway analysis of differentially expressed genes of F1 strain gown with glutamate instead of ammonium


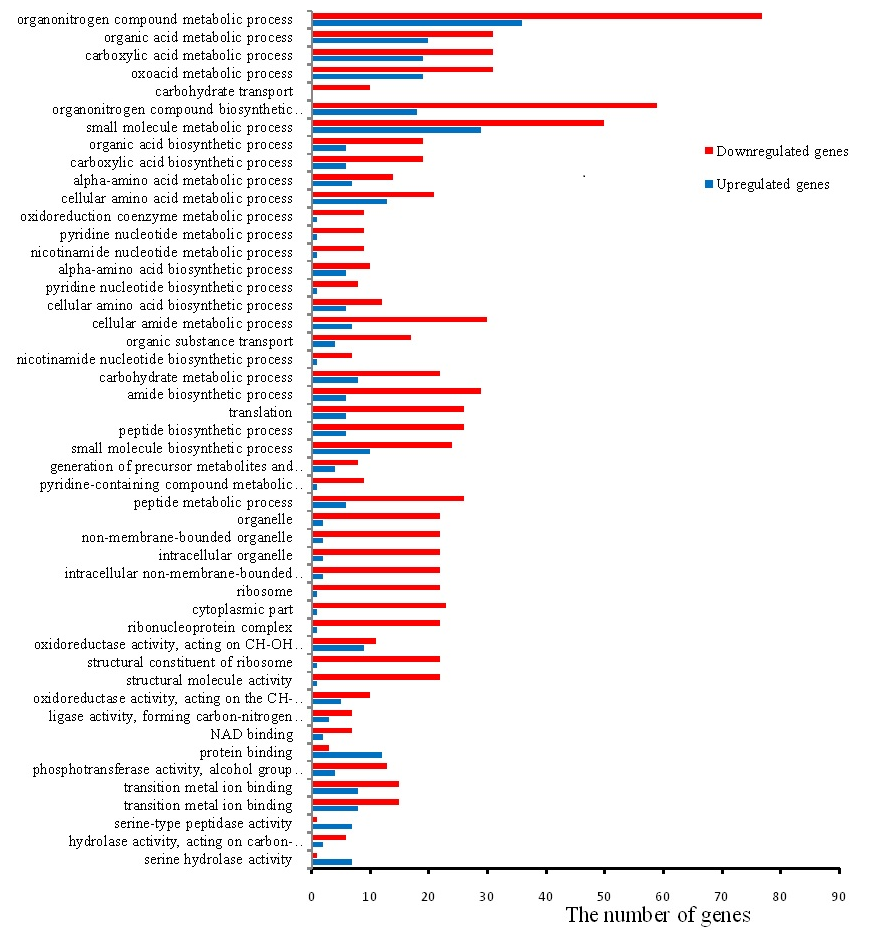


Fig.S4 The Gene-Ontology terms and pathway enrichment analysis of differentially expressed genes of strains F1 vs F5 grown with ammonium


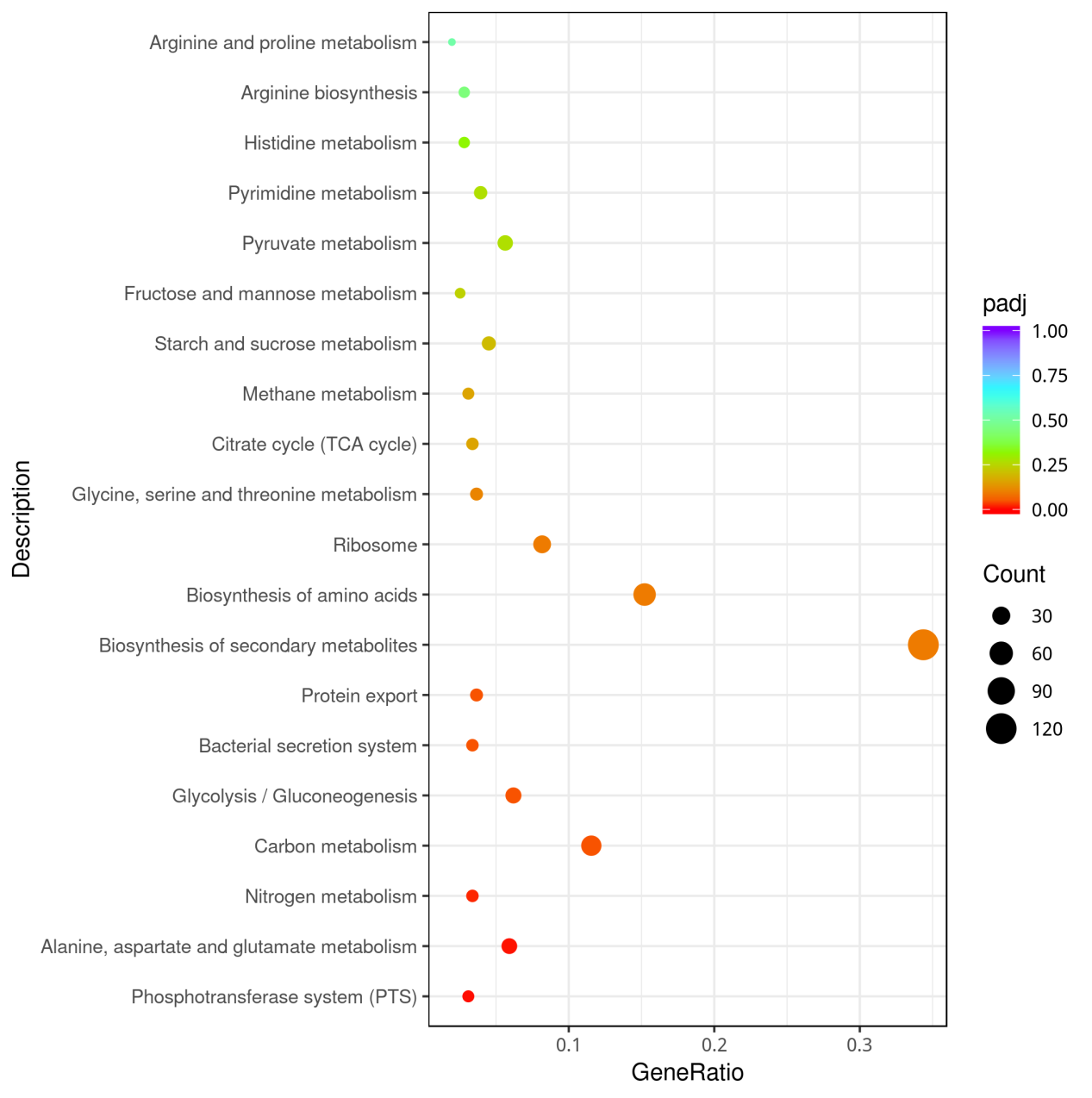


Fig.S5 The KEGG pathway analysis of differentially expressed genes of strains F5 vs F1 grown with ammonium

Fig.S6 The KEGG pathway analysis of differentially expressed genes of strains F3 vs F1 grown with ammoniumig.S6 The KEGG pathway analysis of differentially expressed genes of strains F3 vs F1 grown with ammonium
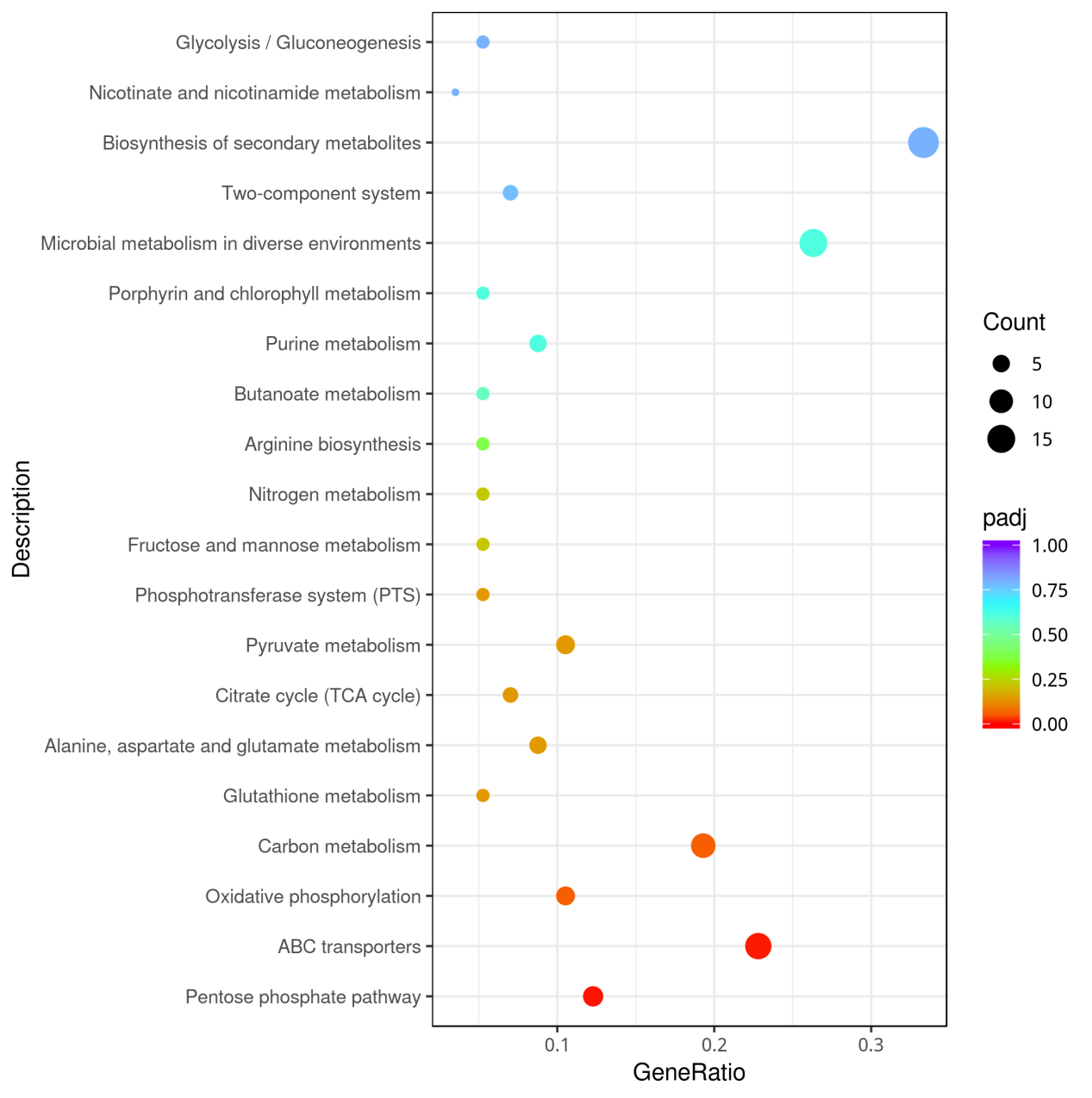


Table S1 The primers are designed in this paper

| Primer name | Sequence(5'-3') |
| --- | --- |
|  |  |
| gdhA F | CGACATATGACAGTTGATGAGCAGGTCTCTAAC |
| gdhA R | CATGAATTCTTAGATGACGCCCTGTGCCAG |
| gdhB F | CAGCATATGTTCGAGCTAATCGACGACTGG |
| gdhB R | CACGAATTCTGTGTTATCGGCGAACCATACCTCTG |
| gdhB-up F | CTGGGATCCACTTGGCGCACACACTCTCCTTGC |
| gdhB-up R | ACTGTCGACTGTCGATGACAAGTACGCCACGCATCC |
| gdhB-dw F | ATGGTCGACACCTCTGAATCTGACCTGACCAGC |
| gdhB-dwR | GTCAAGCTTAGTCATCATTGGAGGCATGGTCGCGC |
| gltBup-F | ACTCCCGGGCCGGAGCAGGTCTTTGATGGCAGTTG |
| gltBup-R | TGCTCTAGACCATCGCCAGTGTTCTTCTCTGCACC |
| gltBdown-F | TGCTCTAGACCACGGTTGTGAGTACATGACTGGC |
| gltBdown-R | GACAAGCTTGACTGATTCTGCACCTTGGCGCAGTG |
| CheckgdhB F | ACTGCGCTTGCCCTGATCTGTGGCG |
| Chec gdhB R | TCTGGCGCGCATAGCTGCAAGAACT |
| CheckgltB F | AAGCATCATCGTTTGGGGTTGGAGG |
| CheckgltB R | GCTCATCAGCCCATGTCAGATCGTC |

Note: The underlined parts in the table are restriction sites.

Table S2 RNA-Sequencing read mapping. L-glutamate (LG) and ammonium sulfate (S) correspond to two different conditions: 50 mL minimal medium plus with 150 mM ammonium sulfate, 70mMl L-glutamate, respectively.

| Sample name | Total reads | Total mapped | Multiple mapped | Uniquely mapped |
| --- | --- | --- | --- | --- |
| F_LG1 | 7458972 | 7346926 (98.5%) | 136194 (1.83%) | 7210732 (96.67%) |
| F_LG2 | 7655750 | 7520441 (98.23%) | 148897 (1.94%) | 7371544 (96.29%) |
| F_LG3 | 7527264 | 7411139 (98.46%) | 143210 (1.9%) | 7267929 (96.55%) |
| FQAB_S1 | 8477954 | 8396661 (99.04%) | 162301 (1.91%) | 8234360 (97.13%) |
| FQAB_S2 | 7591890 | 7511266 (98.94%) | 138713 (1.83%) | 7372553 (97.11%) |
| FQAB_S3 | 7638924 | 7557299 (98.93%) | 161206 (2.11%) | 7396093 (96.82%) |
| FQB_S1 | 8652742 | 8564639 (98.98%) | 153348 (1.77%) | 8411291 (97.21%) |
| FQB_S2 | 7788252 | 7704029 (98.92%) | 152998 (1.96%) | 7551031 (96.95%) |
| FQB_S3 | 7395054 | 7312795 (98.89%) | 143773 (1.94%) | 7169022 (96.94%) |
| F_S1 | 8208712 | 8129097 (99.03%) | 150269 (1.83%) | 7978828 (97.2%) |
| F_S2 | 8190028 | 8098446 (98.88%) | 152294 (1.86%) | 7946152 (97.02%) |
| F_S3 | 7583692 | 7494193 (98.82%) | 164006 (2.16%) | 7330187 (96.66%) |

Note: F_LG1、F_LG2、F_LG3 refer to three biological repetitions of the *C.glutamicum* strain F343 grown with glutamate; F_S1、F_S2、F_S3 refer to the *C.glutamicum* strain F343 grown with ammonium; FQB_S1、FQB_S2、FQB_S3 are the strain *gdhB* deletion mutants grown with ammonium; FQAB_S1、FQAB_S2、FQAB_S3 are the strain *gdhAgdhB* double deletion mutants grown with ammonium;

| Table S3 Statistics of differentially expressed genes with different screening thresholds | | | | | | | | | |
| --- | --- | --- | --- | --- | --- | --- | --- | --- | --- |
|  | padj<0.05 \|log2FC\|>0.0 | | | padj<0.05 \|log2FC\|>0.5 | | | padj<0.05 \|log2FC\|>1 | | |
| Compare | All | Up | Down | All | Up | Down | All | Up | Down |
| FQB_SvsF_S | 142 | 86 | 56 | 141 | 85 | 56 | 89 | 50 | 39 |
| FQAB_SvsF_S | 918 | 472 | 446 | 857 | 444 | 413 | 419 | 252 | 167 |
| F_LGvsF_S | 1833 | 904 | 929 | 1769 | 885 | 884 | 1227 | 661 | 566 |
|  |  |  |  |  |  |  |  |  |  |

Table S4 Description of genes in which response to nitrogen form

| **gene_name** | **gene_description** | | | | |  |
| --- | --- | --- | --- | --- | --- | --- |
| *gluA* | glutamate ABC transporter substrate-binding protein | | | |  |  |
| *gluB* | glutamate ABC transporter substrate-binding protein | | | |  |  |
| *gluC* | amino acid ABC transporter permease | | |  |  |  |
| *gluD* | amino acid ABC transporter permease | | | |  |  |
| *urtA* | urea ABC transporter ATP-binding protein UrtA | | | |  |  |
| *urtB* | urea ABC transporter permease subunit UrtB | | | |  |  |
| *urtC* | urea ABC transporter permease subunit UrtC | | | |  |  |
| *urtD* | urea ABC transporter ATP-binding protein UrtD | | | |  |  |
| *urtE* | urea ABC transporter ATP-binding protein UrtE | | | |  |  |
| *narK* | NarK/NasA family nitrate transporter | | |  |  |  |
| *narG* | nitrate reductase subunit alpha | | | |  |  |
| *narH* | nitrate reductase subunit beta | | | |  |  |
| *narI* | respiratory nitrate reductase subunit gamma | | | |  |  |
| *narJ* | nitrate reductase molybdenum cofactor assembly chaperone | | | |  |  |
| *gdhB* | Glu dehydrogenase | | | |  |  |
| *gdhA* | glutamate dehydrogenase | | | |  |  |
| *gltB* | glutamate synthase large subunit | | | |  |  |
| *gltD* | glutamate synthase subunit beta | | | |  |  |
| *glnA* | Glutamine synthetase | | | |  |  |
| *amtB* | ammonium transporter | | | |  |  |
| *amtA* | ammonium transporter | | | |  |  |
| *glnD* | protein-PII uridylyltransferase | | | |  |  |
| *glnK* | P-II family nitrogen regulator | | | |  |  |
| *gabT* | 4-aminobutyrate--2-oxoglutarate transaminase | | | |  |  |
| *ndh* | NAD(P)/FAD-dependent oxidoreductase | | | |  |  |
| *sdhC* | succinate dehydrogenase | | | |  |  |
| *sdhA* | fumarate reductase | | | |  |  |
| *sdhB* | succinate dehydrogenase cytochrome b subunit | | | |  |  |
| *ppa* | inorganic diphosphatase | | | |  |  |
| *ctaC* | cytochrome c oxidase subunit II | | | |  |  |
| *ctaE* | heme-copper oxidase subunit III | | | |  |  |
| *atpB* | ATP synthase subunit A | | | |  |  |
| *atpA* | ATP synthase subunit alpha | | | |  |  |
| *atpF* | ATP synthase subunit B | | | |  |  |
| *atpH* | ATP synthase subunit delta | | | |  |  |
| *atpE* | ATP synthase F0 subunit C | | | |  |  |
| *gltX* | glutamate--tRNA ligase | | | |  |  |
| *gltX*  *hemA* | tRNA glutamyl-Q(34) synthetase GluQRS  glutamyl-tRNA reductase | | | |  |  |
| *hemL* | glutamate-1-semialdehyde | | | |  |  |
| *hemB* | porphobilinogen synthase | | | |  |  |
| *hemC* | hydroxymethylbilane synthase | |  |  |  |  |
| *hemD* | protoporphyrinogen oxidase | | | |  |  |
| *hemE* | uroporphyrinogen decarboxylase | | | |  |  |
| *hemY* | protoporphyrinogen oxidase | | | |  |  |
| *hemH* | ferrochelatase | | | | | |
| *ctaA* | heme A synthase | | | | | |
| *ChlI* | Mg-chelatase subunit ChlI | | | |  |  |
| *argB* | acetylglutamate kinase | | | |  |  |
| *argC* | N-acetyl-gamma-glutamyl-phosphate reductase | | | |  |  |
| *argD* | acetylornithine transaminase | | | |  |  |
| *argJ* | glutamate N-acetyltransferase | | | |  |  |
| *argF* | ornithine carbamoyltransferase | | | |  |  |
| *ureA* | urease subunit gamma | | | |  |  |
| *ureB* | urease subunit beta | | | |  |  |
| *ureC* | urease subunit alpha | | | |  |  |
| *ureE* | urease accessory protein UreE | | | |  |  |
| *ureF* | urease accessory protein UreF | | | |  |  |
| *ureG* | urease accessory protein UreG | | | |  |  |
| *ureD* | urease accessory protein UreD | | | |  |  |
| *carA* | carbamoyl-phosphate synthase small subunit | | |  |  |  |
| *carB* | carbamoyl-phosphate synthase large subunit | | | |  |  |
| *ilvB* | acetolactate synthase large subunit | | | |  |  |
| *ilvC* | ketol-acid reductoisomerase | | | |  |  |
| *ilvD* | dihydroxy-acid dehydratase | | | |  |  |
| *leuA* | 2-isopropylmalate synthase | | | |  |  |
| *ilvE* | branched-chain amino acid aminotransferase | | | |  |  |
| *pstG(gluc)* | PTS glucose transporter subunit IIA | | | |  |  |
| *ptsP* | phosphoenolpyruvate-protein phosphotransferase | | | |  |  |
| *ptsH* | HPr family phosphocarrier protein | | | |  |  |
| *sugR* | DeoR transcriptional regulator | | | |  |  |
| *fruR* | DeoR transcriptional regulator | | | |  |  |
| *ptsS* | sucrose PTS beta-glucoside transporter subunit IIBCA | | | |  |  |
| *ptsF* | FRUCTOSE PTS sugar transporter subunit IIA | | | |  |  |
| *folE* | GTP cyclohydrolase I FolE | | | |  |  |
| *folK* | 6-hydroxymethyldihydropteridine diphosphokinase | | | |  |  |
| *folC;* | folylpolyglutamate synthase/dihydrofolate synthase | | | |  |  |
| *folP* | dihydropteroate synthase | | | |  |  |
| *folP* | dihydropteroate synthase | | | |  |  |
| *folA;* | dihydrofolate reductase |  |  |  |  |  |
| *folB* | dihydroneopterin aldolase | | | |  |  |
| *hisI* | phosphoribosyl-AMP cyclohydrolase | | | |  |  |
| *hisA* | imidazole-4-carboxamide isomerase | | | |  |  |
| *hisH* | imidazole glycerol phosphate synthase subunit HisH | | | |  |  |
| *hisE* | imidazole glycerol phosphate synthase subunit HisF | | | |  |  |
| *hisB* | imidazoleglycerol-phosphate dehydratase HisB | | | |  |  |
| *hisF* | imidazole glycerol phosphate synthase subunit HisF | | | |  |  |
| *hisC* | histidinol-phosphate transaminase | | | |  |  |
| *hisN* | histidinol-phosphatase | | | |  |  |
| *hisD* | histidinol dehydrogenase | | | |  |  |

Table S5 Description of genes in which response to *gdh* gene deletion

| **gene_name** | **gene_description** |
| --- | --- |
| *glnA* | Glutamine synthetase |
| *argF* | Aspartate carbamoyltransferase |
| *argB* | acetylglutamate kinase |
| *purQ* | phosphoribosylformylglycinamidine synthase subunit |
| *argC* | N-acetyl-gamma-glutamyl-phosphate reductase |
| *argD* | acetylornithine transaminase |
| *argJ* | glutamate N-acetyltransferase |
| *ureC* | urease subunit alpha |
| *ureB* | urease subunit beta |
| *ureA* | urease subunit gamma |
| *cpsA* | carbamoyl-phosphate synthase large subunit |
| *pyc* | pyruvate carboxylase |
| *sdhA* | succinate dehydrogenase flavoprotein subunit |
| *sdhC* | succinate dehydrogenase |
| *sdhB* | succinate dehydrogenase cytochrome b subunit |
| *scot* | acetyl-CoA hydrolase |
| *cs* | citrate synthase |
| *aceE* | pyruvate dehydrogenase |
| *acnA* | aconitate hydratase |
| *pck* | phosphoenolpyruvate carboxykinase |
| *fumC* | fumarate hydratase |
| *idh* | NADP-dependent isocitrate dehydrogenase |
| *mqo* | malate dehydrogenase |
| *sucC* | ADP-forming succinate-CoA ligase subunit beta |
| *sucD* | succinate-CoA ligase subunit alpha |
| *aals* | aspartate ammonia-lyase |
| *gap* | glyceraldehyde-3-phosphate dehydrogenase |
| *pgk* | phosphoglycerate kinase |
| *fbaA* | fructose-bisphosphate aldolase |
| *ldh* | L-lactate dehydrogenase |
| *6pfk* | 6-phosphofructokinase |
| *eno* | phosphopyruvate hydratase |
| *pyk* | pyruvate kinase |
| *g6pi* | glucose-6-phosphate isomerase |
| *aceE* | pyruvate dehydrogenase |
| *pgm* | phosphoglyceromutase |
| *gltB* | glutamate synthase large subunit |
| *gltD* | glutamate synthase subunit beta |
| *glnA* | glutamate-ammonia ligase |
| *gdhA* | NADP-specific glutamate dehydrogenase |
| *amtB* | ammonium transporter |
| *amtA* | ammonium transporter |
| *glnD* | [protein-PII] uridylyltransferase |
| *glnK* | P-II family nitrogen regulator |
| *gnk* | gluconokinase |
| *gndA* | phosphogluconate dehydrogenas |
| *zwf* | glucose-6-phosphate 1-dehydrogenase |
| *tkt* | transketolase |
| *rpi* | ribose 5-phosphate isomerase B |
| *rbsK1* | ribokinase |
| *cydA* | cytochrome bd ubiquinol oxidase subunit I |
| *cydB* | cytochrome bd ubiquinol oxidase subunit II |
| *ctaB* | heme o synthase |
| *ctaA* | heme a synthase |
| *sugR* | DeoR/GlpR transcriptional regulator |
| *fuR* | DeoR/GlpR transcriptional regulator |
| *ptsS* | PTS beta-glucoside transporter subunit IIBCA |
| *pstF* | PTS sugar transporter subunit IIA |

Table S6 The differentially expressed genes involved in metabolism by comparative transcriptomic analysis of strains F3 vs F1

| Gene ID | Gene name | Annotaion | FQB_S  RPKM | F_S  RPKM | Log2FoldChange |
| --- | --- | --- | --- | --- | --- |
| Genes in the pentose phosphate pathway | | | | | |
| *C629_RS12390* | *gnk* | gluconokinase | 47.94 | 17.962 | 1.41 |
| *C629_RS08140* | *gndA* | 6-phosphogluconate dehydrogenase | 8857.94 | 6237.22 | 0.51 |
| *C629_RS08735* | *zwf* | glucose-6-phosphate 1-dehydrogenase | 1063.92 | 590.12 | 0.85 |
| *C629_RS08725* | *tkt* | transketolase | 3175.62 | 2082.42 | 0.61 |
| *C629_RS11985* | rpi | ribose 5-phosphate isomerase B | 1124.01 | 544.87 | 1.04 |
| *C629_RS07705* | rbsK1 | ribokinase | 432.404 | 187.91 | 1.20 |
| Genes involved in the oxidative phosphorylation | | | | | |
| *C629_RS06615* | *cydA* | cytochrome oxidase subunit I | 869.57 | 318.77 | 1.45 |
| *C629_RS06610* | *cydB* | cytochrome oxidase subunit II | 405.76 | 142.92 | 1.51 |
| *C629_RS08720* | *ctaB* | heme o synthase | 346.99 | 148.83 | 1.22 |
| *C629_RS08705* | *ctaA* | heme a synthase | 1140.85 | 621.15 | 0.88 |
| *C629_RS02270* | *sdhA* | succinate dehydrogenase A | 1173.06 | 768.89 | 0.61 |
| *C629_RS02275* | *sdhB* | succinate dehydrogenase B | 523.31 | 302.97 | 0.79 |
| Genes involved in heavy metal resistance | | | | | |
| *C629_RS00610* | - | multicopper oxidase | 0 | 112.12 | -9.28 |
| *C629_RS00615* | - | metal-binding protein | 0 | 61.75 | -8.42 |
| *C629_RS00620* | - | response regulator factor | 0 | 422.46 | -11.20 |
| *C629_RS00625* | - | histidine kinase | 0 | 322.52 | -10.81 |
| *C629_RS00630* | - | heavy-metal-associated domain-containing protein | 0 | 69.14 | -8.59 |
| *C629_RS00635* | - | copper-translocating P-type ATPase | 0 | 695.34 | -10.95 |
| *C629_RS00640* | - | YdhK family protein | 0 | 273.72 | -10.57 |
| *C629_RS00645* | - | Lrp ligand binding domain-containing protein | 0 | 12.12 | -6.077 |
| *C629_RS00655* | - | hypothetical protein | 0 | 21.048 | -6.87 |
| *C629_RS00665* | - | hypothetical protein | 0 | 42.94 | -7.90 |
| *C629_RS00670* | - | recombinase family protein | 0 | 74.42 | -8.69 |
| *C629_RS00675* | - | metalloregulator ArsR transcription factor | 0 | 102.99 | -9.16 |
| *C629_RS00680* | - | cadmium resistance transporter | 0 | 149.33 | -9.709 |
| *C629_RS00685* | merA | mercury(II) reductase | 0 | 76.17 | -8.73 |
| *C629_RS00690* | - | heavy metal-responsive transcriptional regulator | 0 | 62.84 | -8.45 |
| *C629_RS00700* | - | helix-turn-helix transcriptional regulator | 0 | 412.70 | -11.16 |
| *C629_RS00705* | - | aldo/keto reductase | 0 | 325.62 | -10.82 |
| *C629_RS00710* | - | hypothetical protein | 0 | 169.55 | -9.88 |
| *C629_RS00715* | - | AAA family ATPase | 0 | 29.42 | -7.35 |
| *C629_RS00720* | mcrC | 5-methylcytosine-specific restriction endonuclease | 0 | 34.28 | -7.57 |
